# Ocular Firework Injuries among Patients Presented to the Emergency Department During Festival Season in a Tertiary Eye Hospital

**DOI:** 10.31729/jnma.8433

**Published:** 2024-02-29

**Authors:** Ashma Manandhar, Tinku Mukharjee, Rajiv Ranjan Karn

**Affiliations:** 1 Vitreo Retinal Department, Biratnagar Eye Hospital, Biratnagar, Morang, Nepal; 2 Optical Department, Biratnagar Eye Hospital, Biratnagar, Morang, Nepal; 3 Research Department, Biratnagar Eye Hospital, Abhibadan Marg, Biratnagar, Morang, Nepal

**Keywords:** *festivals*, *injuries*, *prevalence*

## Abstract

**Introduction:**

Fireworks can cause severe ocular injuries which can be prevented if used with proper precautions. It causes not only mechanical injuries but also thermal and chemical injuries. This study aimed to find out the prevalence of ocular firework injuries among patients presented to the emergency department during festival season in a tertiary eye hospital.

**Methods:**

This is a descriptive cross-sectional study done among patients presenting in the emergency department of a tertiary eye hospital after obtaining ethical approval from the Institutional Review Committee. Data of patients from medical records between 26 October 2021 to 28 November 2021 and 15 October 2022 to 17 November 2022 was collected. A convenience sampling method was used. The point estimate was calculated at a 95% Confidence Interval.

**Results:**

Among 132 patients, the prevalence of ocular firework injuries was seen in 73 (55.30%) (46.82-63.78, 95% Confidence Interval). Closed globe injury was mostly observed in 56 (76.71%) patients with ocular injuries. The most common age group affected was those less than 30 years old 54 (73.97%).

**Conclusions:**

The prevalence of ocular firework injuries was found to be lower than other studies done in similar settings. Protective measures should be used to prevent ocular injuries. A public awareness program needs to be launched before such festivals.

## INTRODUCTION

Fireworks are used during various events around the world as they enhance the festive mood.^1^ In a systematic review of firework-related ocular injuries, ocular trauma was found in 21.8% (range 16-45%).^2^ These can be dangerous if not used with proper precautions.^3^ It causes not only thermal but also chemical and mechanical injuries.^4^ Trying to check the unlit cracker and lighting it without protective wear can lead to serious consequences.^5^

In developing and developed countries it contributed to 22% of total eye injuries.^2^ These injuries can create heavy economic, medical, and social burdens.^2,6,7^

This study aimed to find out the prevalence of ocular firework injuries among patients presented to the emergency department during festival season in a tertiary eye hospital.

## METHODS

This descriptive cross-sectional study was conducted among patients presented to the Emergency Department at Birtanagar Eye Hospital, Biratnagar, Morang, Nepal. Ethical approval was taken from the Institutional Review Committee (Reference number: BEH IRC-171/A). Data of patients from medical records between 26 October 2021 to 28 November 2021 and 15 October 2022 to 17 November 2022 was collected. All patients during the study time presenting to the Emergency Department of Biratnagar Eye Hospital were included in the study. Patients who had treatment started from other centres were excluded from the study. A convenience sampling method was used. The sample size was calculated by using the following formula:


$$\begin{array}{ll} \text{n} & ={\text{Z}}^{2}\times \frac{\text{p}\times \text{q}}{{\text{e}}^{\text{2}}}\\ & =1.96^{2}\times \frac{0.50\times 0.50}{0.09^{2}}\\ & =119 \end{array}$$

Where,

n = minimum required sample size
Z = 1.96 at 95 % Confidence Interval (CI)
p = prevalence taken as 50% for maximum sample size
q = 1-p
e = margin of error, 9%

The minimum required sample size was 119. However, the final sample size taken was 132.

All patients underwent comprehensive ophthalmic examination. All relevant data related to age, gender, laterality, type of firework, whether the patient was an active ignitor or bystander, baseline visual acuity (VA), details of injuries, diagnosis, whether the patient received surgical and/or medical treatment and final best-corrected VA (BCVA) at the 1 month follow up was recorded. The immediate management comprised of thorough eye wash with normal saline, removal of dust, soot particles and debris and finally ruling out open globe injury. Depending on the severity of the ocular injury, patients were started on copious lubricants, cycloplegics, topical antibiotics and steroid eye drops. All the ocular injuries were classified according to the Birmingham Eye Trauma Terminology System (BETTS) (2004).^8^

Data were entered and analyzed using IBM SPSS Statistics version 17.0. The point estimate was calculated at a 95% CI.

## RESULTS

Among 132 patients, ocular firework injuries were seen in 73 (55.30%) (46.82-63.78, 95% CI) patients. The right eye was involved in 58 (79.45%) of the patients. No bilateral case of ocular injury was noted in our patients. A total of 55 (75.34%) patients were male. The most common age group was 10-30 years seen in 59 (67.12%) (Table 1).

**Table 1:** Age of presentation and types of injuries among patients with firework-related injuries (n = 73).

Age (years)	Open globe injury n (%)	Closed globe injury n (%)	Total n (%)
<10	2 (2.74)	3 (4.11)	5 (6.84)
10-30	12 (16.44)	37 (50.68)	49 (67.12)
31-50	3 (4.11)	13 (17.81)	16 (21.91)
>50	-	3 (4.11)	3 (4.11)

Bystanders and patients not actively involved in igniting the fireworks at the time of ocular injury were 57 (78.08%). The majority of patients with ocular injuries needed medical management 39 (53.42%). Close-globe injuries were noted n 46 (82.14%) patients. Most common injuries were seen in the anterior segment of which corneal abrasion was the commonest 41 (56.16%) (Table 2).

**Table 2:** Ocular injury among patients with firework-related injuries (n = 73).

Anatomical parts of eyes	n (%)
**Appendages of eye**	
Eyelid laceration	16 (21.92)
Eyelash/Eyelid burn	12 (16.44)
**Anterior segment**	
Corneal abrasion	41 (56.16)
Iridocyclitis	34 (46.58)
Hyphaema	22 (30.14)
Corneal perforation	16 (21.92)
Traumatic cataract	19 (26.03)
Scleral perforation	4 (5.48)
Iridodialysis	3 (4.11)
Dislocated lens	1 (1.37)
**Posterior segment**	
Vitreous haemorrhage	21 (28.77)
Retinal detachment	8 (10.96)
Macular hole	4 (5.48)
Intraocular foreign body	1 (1.37)

**Figure 1:**
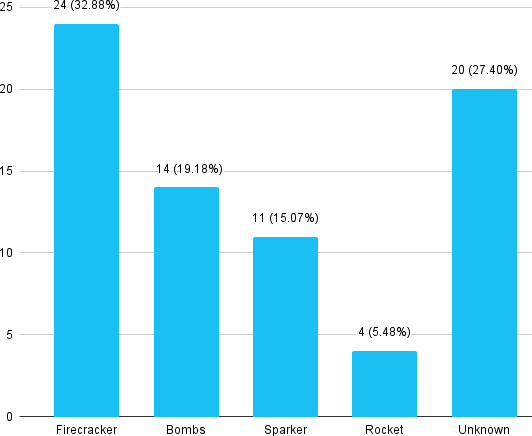
Types of fireworks among patients with ocular firework injuries (n= 73).

Patients with ocular injuries who presented to the hospital within 24 hours were 14 (19.18%). Firecracker was the most common firework causing ocular injury 24 (32.88%) in our study (Figure 1).

Visual acuity of most of the patients 40 (54.79%) on presentation had vision less than 3/60 (Table 3).

**Table 3:** Visual acuity at presentation of patients with ocular firework injuries (n = 73).

Vision	n (%)
6/6-6/9	-
6/12-6/18	5 (6.85)
6/24-6/60	20 (27.40)
5/60-3/60	8 (10.96)
<3/60	40 (54.79)

## DISCUSSION

In our study, the prevalence of ocular firework injuries were seen in 73 (55.30%). In a similar study prevalence of ocular injuries was found to be 65%.^9^ In this study, close-globe injuries were seen in 56 (76.71%) patients.

A total of 55 (75.34%) patients were male similar to the other studies from various parts of the world.^2,5,10,11^ This indicates that men are more prone to eye injuries caused by fireworks because they are more frequently exposed to outdoor activities than females. In our study, 67.12% of the patients were under 30 yrs which is similar to the other study.^2,11^ The possible reason for the lower number of female patients might be because they are involved in indoor activities like decoration and cooking, also parents are more vigilant for firework-related injuries to them because of cosmetic reasons.^12^

In our study, firework-related injury showed a decrease in number as the age increased, mostly because adults are more responsible while handling firecrackers and they are less involved in burning fireworks.^3^ The type of fireworks involved also determines the severity of ocular injury sustained. The majority of injuries in our study were caused by firecrackers accounting for 32.88%, similar to the other study.^9^ The possible reason might be that it is small so it can be easily smuggled through the open border of neighbouring countries as firework is legally banned in Nepal. So, the chances of illegal import of heavy and powerful fireworks is less and so are their injuries.

Eyelid injuries were seen in 38.35% and corneal abrasion was the most common ocular injury noted accounting for 56.16%% higher than previous study.^3^ Open globe injury in our study was 46.5% which was similar to another study (35.5%).^13^ The possible reason for the high number of open globe injuries was that our hospital is a non-profitable tertiary eye hospital which is run by NNJS (Nepal Netra Jyoti Sangh) where most of the referred patient is treated. Most of the open globe injuries in our study occurred during the Chaat festival (64.7%), which is celebrated at a nearby river bank or pond worshipping the sun and has strict rituals. Because the parents are usually busy with the rituals, the children are not under supervision in the crowded area where fireworks are launched, so the chances of trauma to them are high. Many Indian studies have shown that more than 85 % of firecracker injuries occurred while children were not under-supervised by adults.^3,12-14^ Studies have shown that bystanders are more at risk of injuries because they are unaware of the fireworks approaching them and can't anticipate the danger.^3^ In our study 78.8% of the patients who had ocular injuries were bystanders and not actively participating in lunching firework similar to previous study.^8^

There are a few limitations in our study. Restricting the cases studied to those that occurred during only two festivals may have resulted in an underestimation of the magnitude of the problem during other period of year.

## CONCLUSIONS

The prevalence of ocular firework injuries was found to be lower than other studies done in similar settings. Protective measures should be used to prevent ocular injuries. A public awareness program needs to be launched before such festivals as well as strengthened education on safety and proper legislation for using fireworks are the key methods to reduce such serious trauma.
